# Distribution of internal medicine rotations among resident physicians in Japan: a nationwide, multicenter, cross-sectional study

**DOI:** 10.1186/s12909-024-05314-4

**Published:** 2024-03-20

**Authors:** Kiyoshi Shikino, Miwa Sekine, Yuji Nishizaki, Yu Yamamoto, Taro Shimizu, Sho Fukui, Kazuya Nagasaki, Daiki Yokokawa, Takashi Watari, Hiroyuki Kobayashi, Yasuharu Tokuda

**Affiliations:** 1 Department of Community-oriented Medical Education, Chiba University Graduate School of Medicine, 1-8-1 Inohana Chu-ou-ku, Chiba, Japan; 2 Department of General Medicine, Chiba University Hospital, Chiba, Japan; 3 Division of Medical Education, Juntendo University School of Medicine, Tokyo, Japan; 4 Division of General Medicine, Center for Community Medicine, Jichi Medical University, Tochigi, Japan; 5 Department of Diagnostic and Generalist Medicine, Dokkyo Medical University Hospital, Tochigi, Japan; 6 Emergency and general Medicine, Kyorin University School of Medicine, Tokyo, Japan; 7 Institute of Medicine, University of Tsukuba, Tsukuba, Ibaraki Japan; 8 Department of Internal Medicine, Mito Kyodo General Hospital, Tsukuba, Japan; 9 General Medicine Center, Shimane University Hospital, Izumo, Japan; 10 Department of Medicine, University of Michigan Medical School, Ann Arbor, MI USA; 12 Muribushi Okinawa Center for Teaching Hospitals, Okinawa, Japan; 11 Tokyo Foundation for Policy Research, Tokyo, Japan

**Keywords:** Clinical rotation, GM-ITE, Internal medicine, Postgraduate clinical training, Residency program

## Abstract

**Background:**

In Japan, postgraduate clinical training encompasses a 2-year residency program, including at least 24 weeks of internal medicine (IM) rotations. However, the fragmented structure of these rotations can compromise the training’s quality and depth. For example, a resident might spend only a few weeks in cardiology before moving to endocrinology, without sufficient time to deepen their understanding or have clinical experience. This study examined current patterns and lengths of IM rotations within the Japanese postgraduate medical system. It scrutinized the piecemeal approach—whereby residents may engage in multiple short-term stints across various subspecialties without an overarching, integrated experience—and explored potential consequences for their clinical education.

**Methods:**

This nationwide, multicenter, cross-sectional study used data from self-reported questionnaires completed by participants in the 2022 General Medicine In-Training Examination (GM-ITE). Data of 1,393 postgraduate year (PGY) one and two resident physicians who participated in the GM-ITE were included. We examined the IM rotation duration and number of IM subspecialties chosen by resident physicians during a 2-year rotation.

**Results:**

Approximately half of the participants chose IM rotation periods of 32–40 weeks. A significant proportion of participants rotated in 5–7 internal medicine departments throughout the observation period. Notable variations in the distribution of rotations were observed, characterized by a common pattern where resident physicians typically spend 4 weeks in each department before moving to the next. This 4-week rotation is incrementally repeated across different subspecialties without a longer, continuous period in any single area. Notably, 39.7% of participants did not undertake general internal medicine rotations. These results suggest a narrowed exposure to medical conditions and patient care practices.

**Conclusions:**

Our study highlights the need to address the fragmented structure of IM rotations in Japan. We suggest that short, specialized learning periods may limit the opportunity to gain broad in-depth knowledge and practical experience. To improve the efficacy of postgraduate clinical education, we recommend fostering more sustained and comprehensive learning experiences.

**Supplementary Information:**

The online version contains supplementary material available at 10.1186/s12909-024-05314-4.

## Background

In recent years, there has been a notable shift towards community care over traditional hospital settings in Japan [1]. This may reflect a response to Japan’s demographic changes, such as its aging population. The evolving nature of patient preferences, alongside a national agenda to enhance accessibility and personalized care in community settings, may also contribute to this trend. These societal factors are critical in understanding the current and future landscape of medical education, which must adapt to meet these changing healthcare needs.

Japan has 82 medical schools where the curriculum spans 6 years, starting immediately after high school. The initial phase of medical education covers a broad range of general studies for the first 2 years, followed by 4 years of focused medical training. Upon graduation, the vast majority of students embark on residency programs that begin with a required 2-year postgraduate residency training [2, 3]. It is noteworthy that about one-third of these graduates opt to pursue a specialization in internal medicine or its related subspecialties. As part of their postgraduate clinical training, which has been compulsory since April 2004, resident physicians undergo a comprehensive 96-week (2-year) residency rotation program [2, 3]. This program is structured to provide essential foundational knowledge, with a minimum of 24 weeks allocated to internal medicine (IM) or general internal medicine (GIM) subspecialties for the acquisition of acute care skills. In addition to these, resident physicians partake in rotations across six other medical domains (surgery, obstetrics and gynecology, pediatrics, psychiatry, emergency medicine, and community-based medicine), each for at least 4 weeks, ensuring a broad clinical exposure. The program’s design also allows for 40 weeks of flexible scheduling, permitting resident physicians to tailor their training to either deepen their expertise in specific areas such as family medicine or hospital medicine, or to further expand their proficiency within the wider field of general internal medicine, ultimately leading to specialty certification [4, 5]. 

IM is a multifaceted specialty requiring an extensive knowledge base and a diverse set of skills. These competencies are traditionally developed through a series of clinical rotations [5, 6]. The structure of these rotations, however, varies significantly across training programs, potentially leading to varied experiences and skill sets among graduates [7]. This variability underscores the importance of a standardized framework to ensure a uniformly comprehensive educational experience in IM. However, there has been no recent analysis of the distribution of IM subspecialties or length of rotations within Japanese residency programs [2]. Given the global trends in medical education that prioritize adaptability and the swift integration of emerging medical knowledge, [8–10] Japan has placed a particularly heightened emphasis on training efficiency within its postgraduate medical education programs. This national focus is in response to the country’s unique healthcare needs, influenced by its aging population and the necessity for rapid dissemination of innovative treatments and technologies. The structure of the IM residency program, which encompasses the entire scope of training across various medical subspecialties, requires a comprehensive review. There is a distinct lack of recent evaluations concerning how these subspecialties are distributed, the duration of each rotation, and the overall coordination of these rotations within the broader framework of the Japanese residency system [11, 12]. The term “IM residency program” refers to the full curriculum designed to prepare physicians in internal medicine, whereas “dedicated IM rotations” pertain to the specific weeks within this curriculum that focus solely on internal medicine [13–15]. 

This study aimed to comprehensively analyze the distribution and duration of IM rotations in Japan, provide a descriptive overview of prevailing patterns and allocations within IM residency programs, and delve into the potential relationship between the characteristics of these rotations and their performance on standardized tests.

## Methods

### Study design

We conducted a nationwide, multicenter, cross-sectional study to analyze the distribution and duration of IM rotations among Japanese resident physicians. By examining the data from resident physicians who participated in the General Medicine In-Training Examination (GM-ITE) at the end of the 2022 academic year, we aimed to describe the current patterns in IM residency training rotations. Additionally, we explored the relationship between rotation characteristics and standardized test performance. This study adhered to the Strengthening the Reporting of Observational Studies in Epidemiology (STROBE) reporting guidelines for cross-sectional studies [16]. 

### Participants and setting

The study included 9,011 resident physicians who worked in 662 medical institutions nationwide and participated in the GM-ITE from January 17 to 30, 2023. Resident physicians who did not agree to participate in the survey or had missing data on the clinical training environment were excluded.

### GM-ITE

The GM-ITE was developed and introduced in 2011, following the same format as the IM In-Training Examination [17, 18]. It has evolved into a nationwide examination in Japan, with over half of all resident physicians participating. The GM-ITE is taken towards the end of both PGY-1 and PGY-2. The GM-ITE has been validated by the Professional and Linguistic Assessments Board as a reliable measure to assess clinical competency [19, 20]. According to the objectives of clinical training, the GM-ITE is divided into four categories (medical interview/professionalism, clinical diagnosis, physical examination/procedure, and disease knowledge). Additionally, the examination includes items regarding clinical fields in which resident physicians have rotated, including IM, surgery, obstetrics and gynecology, pediatrics, psychiatry, emergency medicine, and community-based medicine. The GM-ITE is not simply a test of medical knowledge but also evaluates resident physicians’ practical skills and management abilities that have been developed in clinical settings. It is conducted via computer-based testing (CBT) and incorporates the advantages of CBT, including video-based questions that test resident physicians’ clinical experience. The primary objective of the GM-ITE is to determine the relative ranking of clinical training facilities in Japan and address their weaknesses in clinical practice. From the perspective of each resident physician, the purpose of taking the GM-ITE is not to pass or fail but rather to establish their own grade ranking in Japan and help them overcome their weaknesses in clinical areas [21]. 

The 2022 GM-ITE included 80 multiple-choice questions regarding medical interviews and professionalism (eight questions), symptomatology and clinical reasoning (18 questions), physical examination and clinical procedures (18 questions), and disease knowledge (36 questions). Each question was worth one point, contributing to a total score of 80. Although the primary language of the examination was Japanese, 12 out of the 80 questions were presented in English, reflecting the importance of bilingual medical competence.

### Data collection

On completing the 2022 GM-ITE, the participants were asked to complete a voluntary questionnaire designed by the Japan Institute for Advancement of Medical Education Program (JAMEP) that included questions regarding postgraduate plans and IM rotation schedules within their residency program. The questions included those about the duration of rotations in various IM departments, as defined by the Japanese Society of IM (GIM, gastroenterology, cardiovascular medicine, endocrinology and metabolism, nephrology, respiratory, hematology, neurology, allergy rheumatology, infectious diseases, and other IM). We surveyed resident physicians about the duration of IM rotations in 4-week intervals. We defined the resident physician popularity index as the product of the number of weeks of IM rotations and the number of participating resident physicians.

The participants also provided their demographic information, including hospital type (community or university), sex, and postgraduate year (PGY; PGY-1 or PGY-2), as well as information regarding their general medicine and IM rotations. The participants were asked about the number of night shifts per month, average number of assigned inpatients, and self-study time per day. The association between the postgraduate clinical training rotation style and 2022 GM-ITE scores was also investigated.

### Statistical analyses

Resident physicians were classified into three groups according to their IM rotation duration: short-term (≤ 32 weeks), intermediate-term (36–48 weeks), and long-term (≥ 52 weeks) groups. The categorization was based on the aim to create equal-sized cohorts for a balanced analysis. We had 11 categories for the rotation length: 24, 28, 32, 36, 40, 44, 48, 52, 56, 60, and 64 weeks. By grouping these into three broader categories, we ensured that each group had a comparable number of participants, thereby facilitating a more uniform distribution for statistical comparison and interpretation. This approach allowed for a more nuanced understanding of the potential impact of rotation length on clinical training outcomes. Data regarding the resident physicians’ IM rotation experience were gathered from a self-reporting questionnaire administered to the resident physicians. In our classification of resident physicians into short-, intermediate-, and long-term IM rotation groups, no categories for 33–35 weeks or 49–51 weeks existed because our institution structures rotations in 4-week units, and as such, the duration of any given rotation will always be a multiple of 4 weeks. This systematic approach to scheduling ensures consistency across different programs and provides a standardized framework for evaluating resident physicians’ experiences and performance in IM rotations.

We conducted multiple pair-wise comparisons of GM-ITE scores among the three IM rotation groups using Tukey’s honestly significant difference test. Resident physicians with missing data for any variable were excluded from the analyses. SPSS Statistics for Windows (version 29.0; IBM, Armonk, NY, USA) was used to conduct statistical analyses, following the STROBE guidelines.

### Ethics

 Informed consent was obtained from all residents who participated in the GM-ITE. The resident physicians were provided the opportunity to opt out of this study. Before the study, a comprehensive explanation of the research, including details regarding data anonymization and voluntary participation, was provided to the participants. This study was conducted according to the principles outlined in the Declaration of Helsinki, and all methodologies adhered to the Ethical Guidelines for Medical and Health Research Involving Human Subjects. The study protocol was approved by the Ethics Review Board of the JAMEP (approval number 23 − 2).

## Results

### Demographics

Overall, 9,011 resident physicians participated in the GM-ITE, including 1,393 from 449 hospitals who consented and were included in the final analysis (response rate: 15.5%). Among these participants, 700 (50.3%) were in PGY-1, and 413 (29.6%) were women (Table 1). Most participants worked in community hospitals (76.7%), and 22.8% worked in university hospitals. The sex ratio was similar between the PGY-1 and PGY-2 groups. Most participants (73.4%) reported three to five night shifts, 9.3% reported six or more night shifts, and 2.2% reported no night shifts. Half of the participants (50.2%) reported managing five to nine inpatients, whereas 37.5% reported managing zero to four inpatients. The mean daily self-study time was 1–30 min in 44.3% of participants and 0 min in 1.7% of participants. Nearly half of the participants (48.7%) reported working < 60 h/week, whereas 14.3% reported working ≥ 80 h.

**Table 1:** Baseline characteristics of resident physicians

		Total	PGY1	PGY2
		n, (%)	n, (%)	n, (%)
		1393 (100)	700 (100)	693 (100)
**GM-ITE score** (Mean, SD)		46.0 ± 8.2	45.1 ± 7.8	46.9 ± 8.5
**Hospital type**				
	Community Hospital	1068 (76.7)	524 (74.9)	544 (78.5)
	University Hospital	318 (22.8)	173 (24.7)	145 (20.9)
**Gender**				
	Women	413 (29.6)	208 (29.7)	205 (29.6)
	Men	980 (70.4)	492 (70.3)	488 (70.4)
**Night shift per month**				
	0	31 (2.2)	19 (2.7)	12 (1.7)
	1–2	192 (13.8)	89 (12.7)	103 (14.9)
	3–5	1023 (73.4)	518 (74.0)	505 (72.9)
	≥ 6	129 (9.3)	66 (9.4)	63 (9.1)
	Unknown	9 (0.6)	3 (0.4)	6 (0.9)
**Average number of assigned inpatients**				
	0–4	522 (37.5)	281 (40.1)	241 (34.8)
	5–9	699 (50.2)	341 (48.7)	358 (51.7)
	10–14	101 (7.3)	42 (6.0)	59 (8.5)
	≥ 15	29 (2.1)	13 (1.9)	16 (2.3)
	Unknown	34 (2.4)	17 (2.4)	17 (2.5)
**Self-study time per day (minutes)**				
	0	23 (1.7)	9 (1.3)	14 (2)
	1–30	617 (44.3)	338 (48.3)	279 (40.3)
	31–60	547 (39.3)	264 (37.7)	283 (40.8)
	61–90	144 (10.3)	64 (9.1)	80 (11.5)
	≥ 91	49 (3.5)	18 (2.6)	31 (4.5)
**Duty-hour per week (hours)**				
	Category 1 (< 60)	679 (48.7)	347 (49.6)	332 (47.9)
	Category 2 (60–79)	501 (36.0)	265 (37.9)	236 (34.1)
	Category 3 (≥ 80)	199 (14.3)	81 (11.6)	118 (17)

GM-ITE: General Medicine In-Training Examination

### IM rotation distribution

**Fig. 1:**
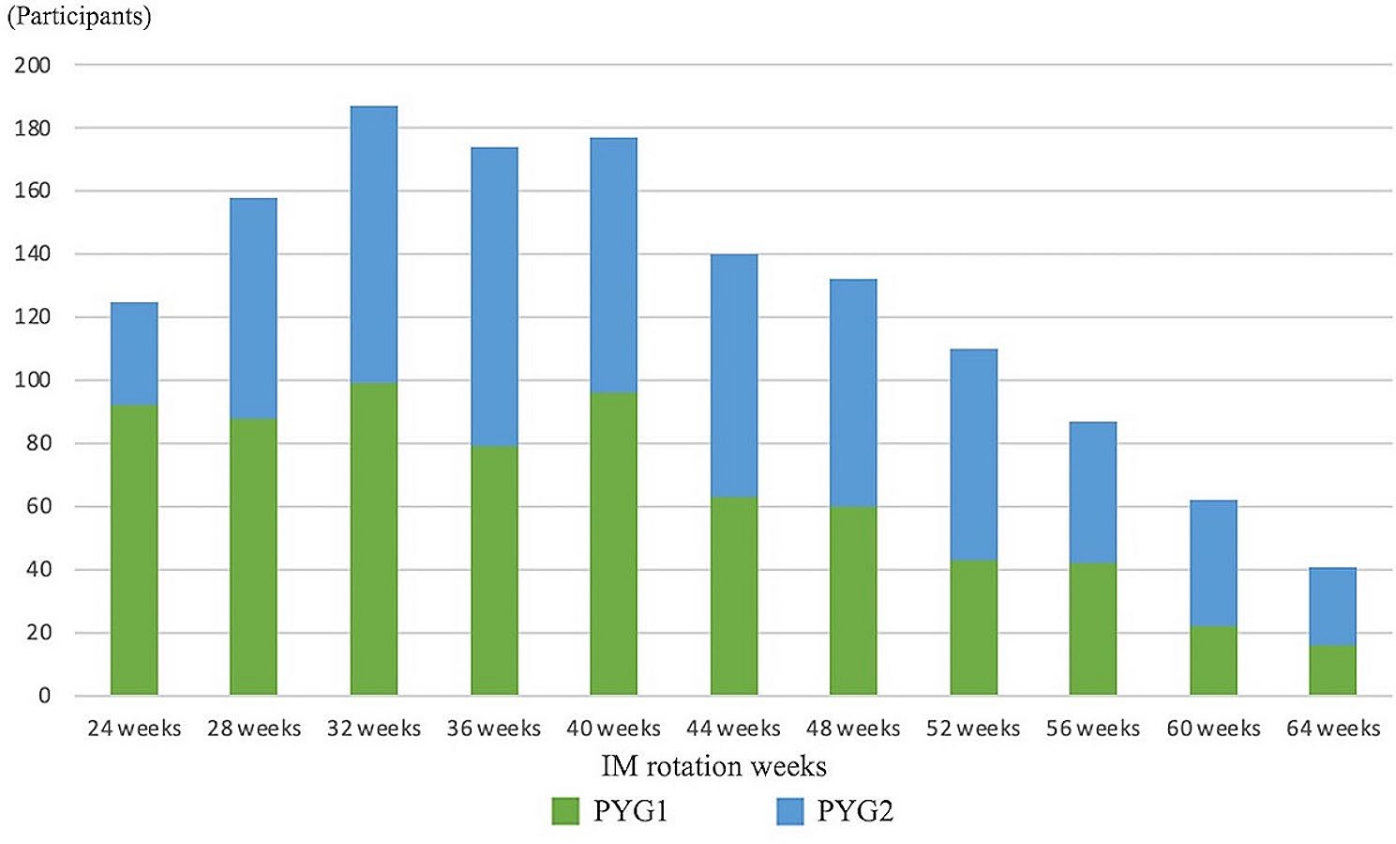
Distribution of the internal medicine total rotation length. This histogram shows the preferred internal medicine rotation lengths on the x-axis and the percentage of residents choosing each length on the y-axis. The most common duration is 32 to 40 weeks, selected by approximately 50% of resident physicians

**Fig. 2:**
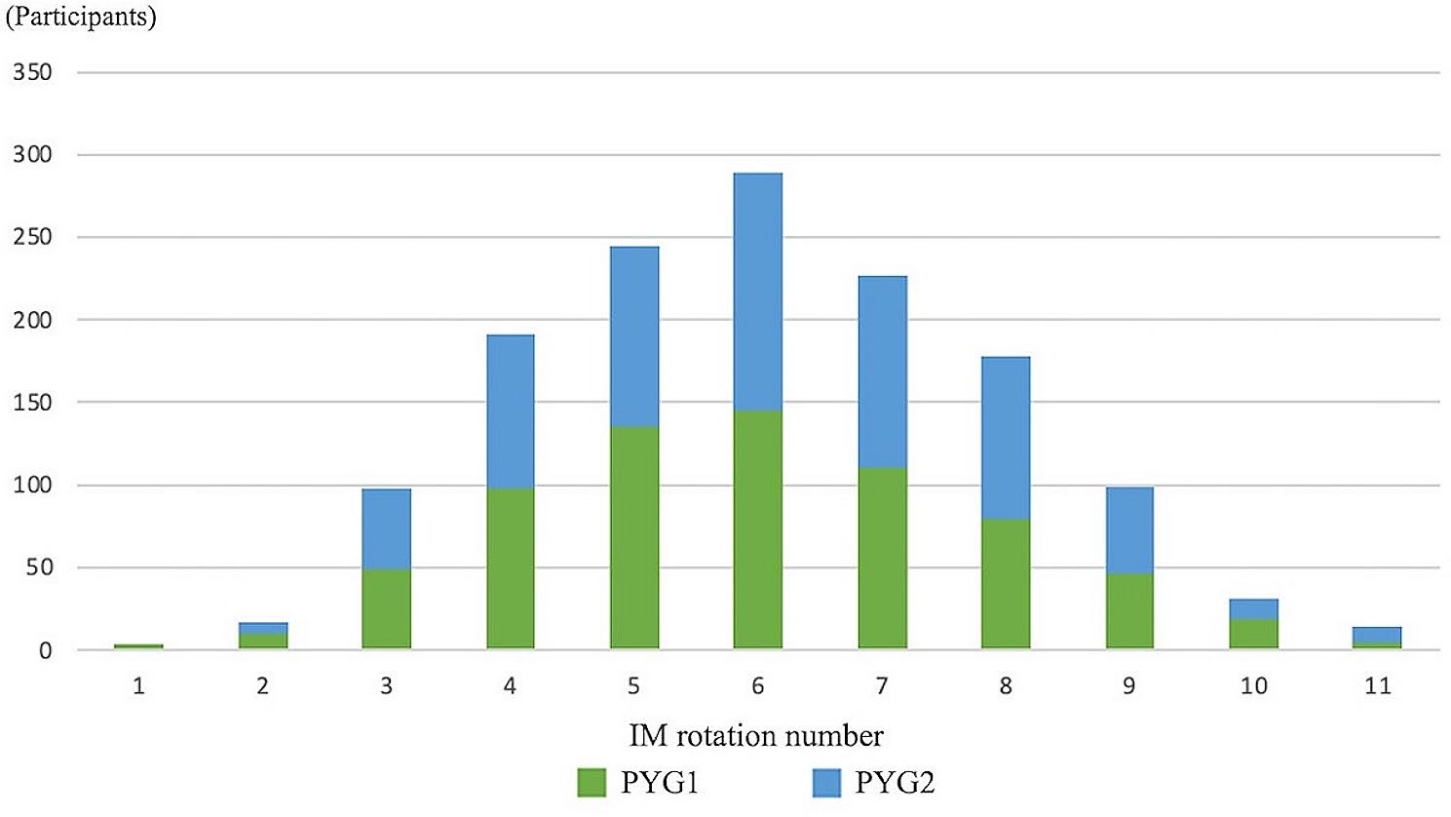
Distribution of the number of internal medicine department rotations. This figure shows the distribution of rotations across internal medicine departments. The x-axis shows the number of departments, and the y-axis shows the percentage of resident physicians. A notable number of resident physicians rotated through five to seven departments

Approximately half of the resident physicians chose IM rotation periods of 32–40 weeks (Fig. 1). A significant proportion of participants rotated in five to seven departments throughout the observation period (Fig. 2). Fewer participants rotated in nine or more departments. The top three IM subspecialties popular among resident physicians were gastroenterology, cardiovascular medicine, and GIM. The resident physician popularity index for gastroenterology, calculated as the product of the number of weeks of IM rotation and number of resident physicians who completed it, was 9,728. A physician popularity index of 9,200 was scored for cardiovascular medicine, while that for general medicine was 7,208 (Table 2 and Supplementary file 1 and 2). It was observed that rotations in 4-week blocks were the most common structure, followed by 8-week rotations, indicating a preference for modular rotation lengths among residency programs.

**Table 2:** Internal medicine rotation distribution in the resident physicians’ clinical training program in Japan

Internal medicine department	Number of resident physicians × weeks (RPP index)		Length of training in each department (n, %)												
Total	Training period	Number	No rotation	4 weeks	8 weeks	12 weeks	16 weeks	20 weeks	24 weeks	28 weeks	32 weeks	36 weeks	40 weeks	44 weeks	48 weeks
Allergy Rheumatology	Total	2180	2354(84.5)	339(12.2) ^*^	79(2.8)	9(0.3)	4(0.1)	1(0.1)	0(0)	0(0)	0(0)	0(0)	0(0)	0(0)	0(0)
	1st year	1304	1149(74.4)	174(11.3) ^*^	60(3.9)	8(0.5)	2(0.1)	0(0)	0(0)	0(0)	0(0)	0(0)	0(0)	0(0)	0(0)
	2nd year	876	1205(78.0)	165(10.7) ^*^	19(1.2)	1(0.1)	2(0.1)	1(0.1)	0(0)	0(0)	0(0)	0(0)	0(0)	0(0)	0(0)
Cardiovascular Medicine	Total	9200	1214(43.6)	948(35.3) ^*^	549(19.7)	59(2.1)	8(0.3)	5(0.1)	2(0.1)	0(0)	1(0.1)	0(0)	0(0)	0(0)	0(0)
	1st year	6640	302(19.6)	583(37.8) ^*^	454(29.4)	50(3.2)	2(0.1)	1(0.1)	1(0.1)	0(0)	0(0)	0(0)	0(0)	0(0)	0(0)
	2nd year	2560	912(59.1)	365(23.6) ^*^	95(6.2)	9(0.6)	6(0.4)	4(0.3)	1(0.1)	0(0)	1(0.1)	0(0)	0(0)	0(0)	0(0)
Endocrinology Metabolism	Total	5360	1709(61.3)	842(30.2) ^*^	213(7.6)	16(0.6)	6(0.2)	0(0)	0(0)	0(0)	0(0)	0(0)	0(0)	0(0)	0(0)
	1st year	3488	718(46.5)	498(32.3) ^*^	161(10.4)	12(0.8)	4(0.3)	0(0)	0(0)	0(0)	0(0)	0(0)	0(0)	0(0)	0(0)
	2nd year	1872	991(64.2)	344(22.3) ^*^	52(3.4)	4(0.3)	2(0.1)	0(0)	0(0)	0(0)	0(0)	0(0)	0(0)	0(0)	0(0)
Gastroenterology	Total	9728	1256(45.1)	890(31.9) ^*^	508(18.2)	70(2.5)	30(1.1)	5(0.1)	20(0.7)	5(0.1)	2(0.1)	0(0)	0(0)	0(0)	0(0)
	1st year	6912	346(22.4)	547(35.4) ^*^	413(26.7)	50(3.2)	10(0.6)	3(0.2)	19(1.2)	4(0.3)	1(0.1)	0(0)	0(0)	0(0)	0(0)
	2nd year	2816	910(58.9)	343(22.2) ^*^	95(6.2)	20(1.3)	20(1.3)	2(0.1)	1(0.1)	1(0.1)	1(0.1)	0(0)	0(0)	0(0)	0(0)
General Internal Medicine	Total	7208	1653(59.3)	696(25.0) ^*^	312(11.2)	75(2.7)	23(0.8)	7(0.3)	16(0.6)	0(0)	2(0.1)	2(0.1)	0(0)	0(0)	0(0)
	1st year	4512	751(48.6)	334(21.6) ^*^	215(13.9)	55(3.6)	16(1)	4(0.3)	15(1)	0(0)	2(0.1)	1(0.1)	0(0)	0(0)	0(0)
	2nd year	2696	902(58.4)	362(23.4) ^*^	97(6.3)	20(1.3)	7(0.5)	3(0.2)	1(0.1)	0(0)	0(0)	1(0.1)	0(0)	0(0)	0(0)
Hematology	Total	3076	2158(77.5)	496(17.8) ^*^	125(4.5)	6(0.2)	0(0)	1(0.1)	0(0)	0(0)	0(0)	0(0)	0(0)	0(0)	0(0)
	1st year	2124	975(63.1)	314(20.3) ^*^	97(6.3)	6(0.4)	0(0)	1(0.1)	0(0)	0(0)	0(0)	0(0)	0(0)	0(0)	0(0)
	2nd year	952	1183(76.6)	182(11.8) ^*^	28(1.8)	0(0)	0(0)	0(0)	0(0)	0(0)	0(0)	0(0)	0(0)	0(0)	0(0)
Infectious Diseases	Total	1056	2555(91.7)	203(7.3) ^*^	25(0.9)	1(0.1)	2(0.1)	0(0)	0(0)	0(0)	0(0)	0(0)	0(0)	0(0)	0(0)
	1st year	416	1311(84.9)	63(4.1) ^*^	17(1.1)	1(0.1)	1(0.1)	0(0)	0(0)	0(0)	0(0)	0(0)	0(0)	0(0)	0(0)
	2nd year	640	1244(80.6)	140(9.1) ^*^	8(0.5)	0(0)	1(0.1)	0(0)	0(0)	0(0)	0(0)	0(0)	0(0)	0(0)	0(0)
Nephrology	Total	5172	1748(62.7)	804(28.9) ^*^	214(7.7)	19(0.7)	1(0.1)	0(0)	0(0)	0(0)	0(0)	0(0)	0(0)	0(0)	0(0)
	1st year	3184	772(50.0)	459(29.7) ^*^	150(9.7)	11(0.7)	1(0.1)	0(0)	0(0)	0(0)	0(0)	0(0)	0(0)	0(0)	0(0)
	2nd year	1988	976(63.2)	345(22.3) ^*^	64(4.1)	8(0.5)	0(0)	0(0)	0(0)	0(0)	0(0)	0(0)	0(0)	0(0)	0(0)
Neurology	Total	5128	1771(63.6)	770(27.6) ^*^	225(8.1)	18(0.6)	2(0.1)	0(0)	0(0)	0(0)	0(0)	0(0)	0(0)	0(0)	0(0)
	1st year	3248	770(49.9)	443(28.7) ^*^	171(11.1)	9(0.6)	0(0)	0(0)	0(0)	0(0)	0(0)	0(0)	0(0)	0(0)	0(0)
	2nd year	1880	1001(64.8)	327(21.2) ^*^	54(3.5)	9(0.6)	2(0.1)	0(0)	0(0)	0(0)	0(0)	0(0)	0(0)	0(0)	0(0)
Respiratory	Total	6836	1495(53.7)	916(32.9) ^*^	340(12.2)	31(1.1)	2(0.1)	0(0)	2(0.1)	0(0)	0(0)	0(0)	0(0)	0(0)	0(0)
	1st year	4744	538(34.8)	551(35.7) ^*^	280(18.1)	23(1.5)	0(0)	0(0)	1(0.1)	0(0)	0(0)	0(0)	0(0)	0(0)	0(0)
	2nd year	2092	957(62.0)	365(23.6) ^*^	60(3.9)	8(0.5)	2(0.1)	0(0)	1(0.1)	0(0)	0(0)	0(0)	0(0)	0(0)	0(0)
Other Internal Medicine	Total	1240	2568(91.2)	144(5.2) ^*^	62(2.2)	8(0.3)	2(0.1)	2(0.1)	0(0)	0(0)	0(0)	0(0)	0(0)	0(0)	0(0)
	1st year	580	1291(83.6)	64(4.1) ^*^	34(2.2)	3(0.2)	1(0.1)	0(0)	0(0)	0(0)	0(0)	0(0)	0(0)	0(0)	0(0)
	2nd year	660	1277(82.7)	80(5.2) ^*^	28(1.8)	5(0.3)	1(0.1)	2(0.1)	0(0)	0(0)	0(0)	0(0)	0(0)	0(0)	0(0)

^*^Rotation period with the largest number of people in each department (excluding no rotation) RPP index: Resident physician popularity index

Among PGY-1 and PGY-2 participants, there was a notable absence of rotations in several subspecialties. Specifically, 59.3% of participants did not have a GIM rotation, while 91.7% of participants lacked an infectious disease rotation. Similarly, there was an absence of rotations in endocrinology metabolism (61.3% of participants), nephrology (62.7% of participants), neurology (63.6% of participants), and respiratory medicine (53.7% of participants). Cardiovascular medicine rotations were missing for 43.6% of participants.

### GM-ITE score

**Fig. 3:**
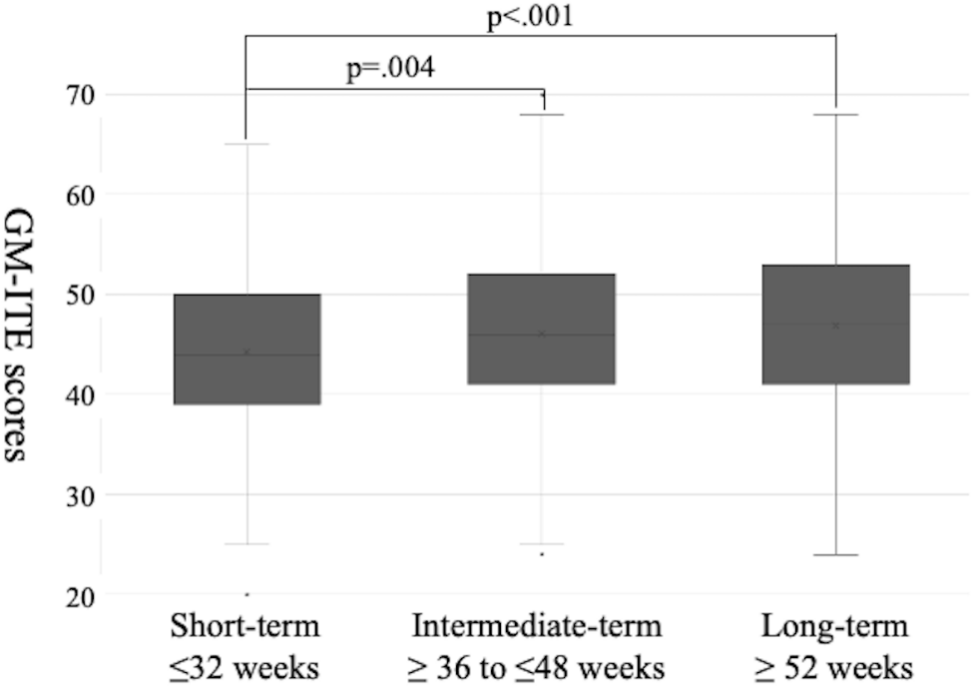
Relationship between the internal medicine rotation length and GM-ITE scores in PGY-2 participants. This figure shows the mean GM-ITE scores for PGY-2 residents across three rotation duration groups: short-term (≤ 32 weeks), intermediate-term (36–48 weeks), and long-term (≥ 52 weeks). Scores increase with longer rotations, from 45.31 in the short-term to 47.69 in the long-term group

The 2022 GM-ITE comprised 80 multiple-choice questions, each valued at one point, yielding a total possible score of 80. The mean GM-ITE score was 45.3 ± 8.5 (95% CI: 44.1–46.5) in the short-term group (*n* = 191), 47.4 ± 8.5 (95% CI: 46.4–48.3) in the intermediate-term group (*n* = 325), and 47.7 ± 8.6 (95% CI: 46.4–48.5) in the long-term group (*n* = 177) (Fig. 3). The short-term group had significantly lower GM-ITE scores than those of the intermediate- (*p* =.004) and long-term groups (*p* <.001). No significant difference existed in GM-ITE scores between the intermediate- and long-term groups (*p* =.24).

## Discussion

As healthcare continues to evolve rapidly, the current structure of postgraduate clinical training in Japan, with its fragmented rotations, is increasingly recognized as insufficient for the development of essential clinical competencies. The evolution and structure of postgraduate clinical training in Japan have been of paramount interest to medical educators, healthcare policymakers, and residency program directors, particularly in their efforts to ensure that resident physicians acquire a comprehensive knowledge base and skill set [22, 23]. This study provides an updated perspective on the current landscape of IM rotations in Japanese residency programs, revealing strengths and areas that require further attention.

One of the most significant findings of the current study was the fragmented nature of IM rotations. Although this structure potentially offers resident physicians exposure to a multitude of specialties, short-duration rotations may hinder in-depth learning and skill acquisition. In the context of Japanese clinical training, the challenge lies not in the 4-week rotation duration itself, which is standard practice, but in the limited availability and potential undervaluation of rotations in GIM. This is compounded by a rotation experience that is often passive and less intensive, which may contribute significantly to the limited depth of clinical exposure and hands-on experience for Japanese residents. It is plausible that the fragmented structure of IM rotations is a significant factor limiting the depth of clinical exposure and hands-on experience for Japanese resident physicians.

A promising approach to mitigate these challenges is integrating general medicine physicians, including primary care physicians and hospitalists, into the educational framework [24, 25] On the basis of their holistic approach, these practitioners can provide comprehensive educational experiences, bridging gaps created by fragmented rotations. Thus, when general medicine physicians educate residents, they can provide them with comprehensive and cross-sectional perspectives, which can fill gaps in fragmented education and contribute to improvements in the learning quality. However, the scarcity of general medicine physicians in Japan poses a challenge in providing sufficient quality and quantity of resident training in general medicine. There is an urgent need to prioritize the cultivation of professionals who can play pivotal roles in enhancing the quality of residency education [26]. 

Furthermore, the increasing popularity of community hospitals and decline in the appeal of university hospitals necessitate the development of more attractive training programs to retain and attract talent [27]. The current study’s findings suggest that re-evaluation and restructuring of the rotation residency program may be beneficial.

### Weaknesses in clinical training during IM rotations

In the Japanese residency program, IM rotations are limited by their fragmented nature. Our nationwide surveys regarding IM rotations are underway, among 1393 resident physicians. More than half (64.7%) of resident physicians in Japan rotate through specialized IM departments every 4 weeks, focusing on specific areas of expertise. When the rotation duration decreases, opportunities for the supervising physician to allow the resident to make independent judgments are limited, leading to more observational training. Resident physicians may have fewer opportunities to understand the pathophysiology of the disease and tend to spend most of their time on tasks such as ordering laboratory tests, as directed by the supervising physician, and documenting the test results in patients’ medical records. Therefore, Japanese resident physicians may have a more passive attitude toward their clinical training than resident physicians in other countries. Moreover, learning the entire field of IM within the 2-year residency period is challenging, especially given that resident physicians may not have the chance to rotate in multiple IM subspecialties. Considering the aim of residency in Japan to instill fundamental clinical skills irrespective of future specialization, dedicating sufficient time to rotations in general medicine, known for its proficiency in handling common diseases, could contribute to acquiring skill sets across various IM areas. During the third wave of the coronavirus disease 2019 (COVID-19) pandemic, a resident physician-based survey was conducted to investigate the actual state of COVID-19 patient care [27]. This previous survey indicated that 47% of resident physicians had no experience in COVID-19 patient care [27]. Furthermore, IM is typically divided into 11 subspecialties, including cardiology, respiratory medicine, gastroenterology, rheumatology, nephrology, neurology, infectious diseases, oncology, hematology, diabetes and endocrinology, and general medicine. However, the most common number of medical specialties reported by participants in this study was six. Thus, within the 2-year rotation framework, resident physicians may not experience certain IM domains due to the rotational structure, which could be compounded by their pursuit of interests in other medical specialties.

In addition to the observational training style, involving watching the supervising physicians engage in actual medical procedures, [28] the structure of short rotation periods in internal medicine also has other limitations. When the environment surrounding resident physicians, including supervising physicians and nurses, undergoes substantial changes, establishing relationships and building trust with supervising physicians requires time, leading to inefficient learning and reduced effectiveness during fragmented rotations. A lower number of supervising physicians is associated with higher GM-ITE scores [29]. 

### Suggestions for overcoming weaknesses

To address limitations identified in our clinical training, a greater involvement of general medicine physicians is essential. These physicians, encompassing a holistic view and managing broad spectrums of conditions, are crucial in imparting comprehensive skill sets to resident physicians. Their expertise in core clinical competencies—such as ethics, communication, examination techniques, symptom analysis, clinical reasoning, and professionalism—is invaluable [30–32]. The United States model, where over 50,000 hospitalists contribute significantly to resident physician education, serves as an exemplar of such an approach. However, Japan faces a shortage of general medicine practitioners, which hinders the promotion of this field among young doctors and resident physicians [33, 34]. 

To enhance resident physicians’ education, it might be beneficial to consider a more active integration of general medicine physicians into the teaching framework [35]. This integration could potentially be facilitated by the establishment of formal mentorship programs, an increase in the number of generalist-led teaching rounds, and offering residents longer rotations in general medicine for a more immersive learning experience [7]. Moreover, Japan’s healthcare system may benefit from incentivizing primary care and hospitalist roles through thoughtful policy changes, examining options for more substantial funding for these positions, and assessing the development of clearer career pathways for resident physicians in these areas [36]. Such exploratory measures could play a role in supporting high-quality internal medicine education and contribute to the evolution of Japan’s clinical training landscape.

### Limitations

This study had several limitations. First, the data used in this study were predominantly self-reported at the conclusion of the GM-ITE. While the data provide a comprehensive overview, there may be inherent biases or inaccuracies in self-reporting, leading to potential discrepancies between reported and actual experiences. Second, the projected rotations are limited. PGY-1 participants reported scheduled rotations, which may change throughout the year. Sudden changes, personal choices, and institutional requirements may lead to deviations from scheduled rotations. Third, the results cannot be generalized easily as only Japan residents were included. However, this study provides crucial insights into the Japanese medical education system. These insights may not be directly applicable to other countries with different training structures and healthcare systems. Fourth, the evolution of training programs cannot be ignored. The current study provides a snapshot of the current state of IM rotations. However, training programs are dynamic and may undergo changes on the basis of feedback, institutional decisions, or policy changes. Fifth, the study did not account for potential confounding variables that could influence GM-IE scores and resident experience. Sixth, the survey had a low response rate; out of 9,011 resident physicians who took the GM-ITE, 1,393 (15.5%) provided responses. This could introduce response bias and affect the representativeness of the results, limiting the generalizability of the findings. Last, owing to the broad study scope, specific subspecialties or nuanced experiences within IM rotations were not thoroughly analyzed. An analysis of these variables may offer further insights regarding the duration and composition of IM rotations in Japan.

## Conclusions

This nationwide, multicenter study provides an exploratory overview of the current IM rotations of resident physicians in Japan, indicating potential areas for improvement. These findings highlight the fragmented structure of IM rotations, characterized by short, specialized periods that might affect the depth of clinical exposure and experiential learning opportunities. This pattern, which primarily consists of 4-week rotations, suggests an inclination toward observational learning, possibly affecting training quality.

### Electronic supplementary material

Below is the link to the electronic supplementary material.

Supplementary Material 1

Supplementary Material 2

## Data Availability

The data sets will not be publicly available because informed consent of participants in each hospital do not allow for such publication. The corresponding author will respond to requests on data analyses.

## Abbreviations

ARCP: Annual Review of Competence Progression
CBT: Computer-based testing
COVID-19: Coronavirus disease 2019
GIM: General internal medicine
GM-ITE: General Medicine In-Training Examination
IM: Internal medicine
JAMEP: Japan Institute for Advancement of Medical Education Program
PGY: Postgraduate year
