# Supplementary material for: Distribution of internal medicine rotations among resident physicians in Japan: a nationwide, multicenter, cross-sectional study
Source: BMC Med Educ. 2024 Mar 20;24:316. doi: 10.1186/s12909-024-05314-4 (PMC10956328; doi:10.1186/s12909-024-05314-4)
Supplement: Supplementary file 1 — Supplementary Material 1 [file 12909_2024_5314_MOESM1_ESM.docx]

| **Internal medicine department** | **Number of resident physicians × weeks (RPP index)** | | **Length of training in each department (n, %)** | | | | | | | | | | | | |
| --- | --- | --- | --- | --- | --- | --- | --- | --- | --- | --- | --- | --- | --- | --- | --- |
| **PGY1** | **Training period** | **Number** | **No rotation** | **4 weeks** | **8 weeks** | **12 weeks** | **16 weeks** | **20 weeks** | **24 weeks** | **28 weeks** | **32 weeks** | **36 weeks** | **40 weeks** | **44 weeks** | **48 weeks** |
| Allergy Rheumatology | Total | 988 | 1207(86.2) | 148(10.6) ^*^ | 38(2.7) | 5(0.4) | 2(0.1) | 0(0) | 0(0) | 0(0) | 0(0) | 0(0) | 0(0) | 0(0) | 0(0) |
|  | 1st year | 620 | 586(38.0) | 80(5.2) ^*^ | 28(1.8) | 5(0.3) | 1(0.1) | 0(0) | 0(0) | 0(0) | 0(0) | 0(0) | 0(0) | 0(0) | 0(0) |
|  | 2nd year | 368 | 621(40.2) | 68(4.4) ^*^ | 10(0.6) | 0(0) | 1(0.1) | 0(0) | 0(0) | 0(0) | 0(0) | 0(0) | 0(0) | 0(0) | 0(0) |
| Cardiovascular Medicine | Total | 4444 | 632(45.1) | 465(33.2) ^*^ | 272(19.4) | 25(1.8) | 3(0.2) | 3(0.2) | 0(0) | 0(0) | 0(0) | 0(0) | 0(0) | 0(0) | 0(0) |
|  | 1st year | 3284 | 162(10.5) | 283(18.3) ^*^ | 230(14.9) | 23(1.5) | 1(0.1) | 1(0.1) | 0(0) | 0(0) | 0(0) | 0(0) | 0(0) | 0(0) | 0(0) |
|  | 2nd year | 1160 | 470(30.4) | 182(11.8) ^*^ | 42(2.7) | 2(0.1) | 2(0.1) | 2(0.1) | 0(0) | 0(0) | 0(0) | 0(0) | 0(0) | 0(0) | 0(0) |
| Endocrinology Metabolism | Total | 2612 | 870(62.1) | 418(29.9) ^*^ | 102(7.3) | 9(0.6) | 1(0.1) | 0(0) | 0(0) | 0(0) | 0(0) | 0(0) | 0(0) | 0(0) | 0(0) |
|  | 1st year | 1732 | 361(23.4) | 253(16.4) ^*^ | 79(5.1) | 6(0.4) | 1(0.1) | 0(0) | 0(0) | 0(0) | 0(0) | 0(0) | 0(0) | 0(0) | 0(0) |
|  | 2nd year | 880 | 509(33.0) | 165(10.7) ^*^ | 23(1.5) | 3(0.2) | 0(0) | 0(0) | 0(0) | 0(0) | 0(0) | 0(0) | 0(0) | 0(0) | 0(0) |
| Gastroenterology | Total | 4576 | 670(47.9) | 428(30.6) ^*^ | 241(17.2) | 36(2.6) | 13(0.9) | 2(0.1) | 7(0.5) | 2(0.1) | 1(0.1) | 0(0) | 0(0) | 0(0) | 0(0) |
|  | 1st year | 3412 | 183(11.9) | 269(17.4) ^*^ | 203(13.1) | 28(1.8) | 5(0.3) | 2(0.1) | 7(0.5) | 2(0.1) | 1(0.1) | 0(0) | 0(0) | 0(0) | 0(0) |
|  | 2nd year | 1164 | 487(31.5) | 159(10.3) ^*^ | 38(2.5) | 8(0.5) | 8(0.5) | 0(0) | 0(0) | 0(0) | 0(0) | 0(0) | 0(0) | 0(0) | 0(0) |
| General Internal Medicine | Total | 3588 | 832(59.4) | 346(24.7) ^*^ | 163(11.6) | 34(2.4) | 13(0.9) | 3(0.2) | 8(0.6) | 0(0) | 1(0.1) | 0(0) | 0(0) | 0(0) | 0(0) |
|  | 1st year | 2264 | 383(24.8) | 157(10.2) ^*^ | 113(7.3) | 26(1.7) | 10(0.6) | 3(0.2) | 7(0.5) | 0(0) | 1(0.1) | 0(0) | 0(0) | 0(0) | 0(0) |
|  | 2nd year | 1324 | 449(29.1) | 189(12.2) ^*^ | 50(3.2) | 8(0.5) | 3(0.2) | 0(0) | 1(0.1) | 0(0) | 0(0) | 0(0) | 0(0) | 0(0) | 0(0) |
| Hematology | Total | 1396 | 1114(79.6) | 225(16.1) ^*^ | 59(4.2) | 2(0.1) | 0(0) | 0(0) | 0(0) | 0(0) | 0(0) | 0(0) | 0(0) | 0(0) | 0(0) |
|  | 1st year | 996 | 503(32.6) | 147(9.5) ^*^ | 48(3.1) | 2(0.1) | 0(0) | 0(0) | 0(0) | 0(0) | 0(0) | 0(0) | 0(0) | 0(0) | 0(0) |
|  | 2nd year | 400 | 611(39.6) | 78(5.1) ^*^ | 11(0.7) | 0(0) | 0(0) | 0(0) | 0(0) | 0(0) | 0(0) | 0(0) | 0(0) | 0(0) | 0(0) |
| Infectious Diseases | Total | 520 | 1290(92.1) | 91(6.5) ^*^ | 18(1.3) | 1(0.1) | 0(0) | 0(0) | 0(0) | 0(0) | 0(0) | 0(0) | 0(0) | 0(0) | 0(0) |
|  | 1st year | 216 | 660(42.7) | 27(1.7) ^*^ | 12(0.8) | 1(0.1) | 0(0) | 0(0) | 0(0) | 0(0) | 0(0) | 0(0) | 0(0) | 0(0) | 0(0) |
|  | 2nd year | 304 | 630(40.8) | 64(4.1) ^*^ | 6(0.4) | 0(0) | 0(0) | 0(0) | 0(0) | 0(0) | 0(0) | 0(0) | 0(0) | 0(0) | 0(0) |
| Nephrology | Total | 2492 | 902(64.4) | 380(27.1) ^*^ | 111(7.9) | 7(0.5) | 0(0) | 0(0) | 0(0) | 0(0) | 0(0) | 0(0) | 0(0) | 0(0) | 0(0) |
|  | 1st year | 1640 | 383(24.8) | 230(14.9) ^*^ | 81(5.2) | 6(0.4) | 0(0) | 0(0) | 0(0) | 0(0) | 0(0) | 0(0) | 0(0) | 0(0) | 0(0) |
|  | 2nd year | 852 | 519(33.6) | 150(9.7) ^*^ | 30(1.9) | 1(0.1) | 0(0) | 0(0) | 0(0) | 0(0) | 0(0) | 0(0) | 0(0) | 0(0) | 0(0) |
| Neurology | Total | 2584 | 896(64.0) | 373(26.6) ^*^ | 122(8.7) | 7(0.5) | 2(0.1) | 0(0) | 0(0) | 0(0) | 0(0) | 0(0) | 0(0) | 0(0) | 0(0) |
|  | 1st year | 1684 | 383(24.8) | 216(14.0) ^*^ | 98(6.3) | 3(0.2) | 0(0) | 0(0) | 0(0) | 0(0) | 0(0) | 0(0) | 0(0) | 0(0) | 0(0) |
|  | 2nd year | 900 | 513(33.2) | 157(10.2) ^*^ | 24(1.6) | 4(0.3) | 2(0.1) | 0(0) | 0(0) | 0(0) | 0(0) | 0(0) | 0(0) | 0(0) | 0(0) |
| Respiratory | Total | 3348 | 763(54.5) | 453(32.4) ^*^ | 171(12.2) | 12(0.9) | 0(0) | 0(0) | 1(0.1) | 0(0) | 0(0) | 0(0) | 0(0) | 0(0) | 0(0) |
|  | 1st year | 2368 | 272(17.6) | 277(17.9) ^*^ | 141(9.1) | 9(0.6) | 0(0) | 0(0) | 1(0.1) | 0(0) | 0(0) | 0(0) | 0(0) | 0(0) | 0(0) |
|  | 2nd year | 980 | 491(31.8) | 176(11.4) ^*^ | 30(1.9) | 3(0.2) | 0(0) | 0(0) | 0(0) | 0(0) | 0(0) | 0(0) | 0(0) | 0(0) | 0(0) |
| Other Internal Medicine | Total | 560 | 1304(93.1) | 59(4.2) ^*^ | 31(2.2) | 5(0.4) | 1(0.1) | 0(0) | 0(0) | 0(0) | 0(0) | 0(0) | 0(0) | 0(0) | 0(0) |
|  | 1st year | 236 | 659(42.7) | 25(1.6) ^*^ | 14(0.9) | 2(0.1) | 0(0) | 0(0) | 0(0) | 0(0) | 0(0) | 0(0) | 0(0) | 0(0) | 0(0) |
|  | 2nd year | 324 | 645(41.8) | 34(2.2) ^*^ | 17(1.1) | 3(0.2) | 1(0.1) | 0(0) | 0(0) | 0(0) | 0(0) | 0(0) | 0(0) | 0(0) | 0(0) |

^*^Rotation period with the largest number of people in each department (excluding no rotation)

**PYG: Postgraduate year**

**RPP index: Resident physician popularity index**
