# Supplementary material for: Distribution of internal medicine rotations among resident physicians in Japan: a nationwide, multicenter, cross-sectional study
Source: BMC Med Educ. 2024 Mar 20;24:316. doi: 10.1186/s12909-024-05314-4 (PMC10956328; doi:10.1186/s12909-024-05314-4)
Supplement: Supplementary file 2 — Supplementary Material 2 [file 12909_2024_5314_MOESM2_ESM.docx]

| **Internal medicine department** | **Number of resident physicians × weeks (RPP index)** | | **Length of training in each department (n, %)** | | | | | | | | | | | | |
| --- | --- | --- | --- | --- | --- | --- | --- | --- | --- | --- | --- | --- | --- | --- | --- |
| **PGY2** | **Training period** | **Number** | **No rotation** | **4 weeks** | **8 weeks** | **12 weeks** | **16 weeks** | **20 weeks** | **24 weeks** | **28 weeks** | **32 weeks** | **36 weeks** | **40 weeks** | **44 weeks** | **48 weeks** |
| Allergy Rheumatology | Total | 1192 | 1147(82.8) | 191(26.5)^*^ | 41(3.0) | 4(0.3) | 2(0.1) | 1(0.1) | 0(0) | 0(0) | 0(0) | 0(0) | 0(0) | 0(0) | 0(0) |
|  | 1st year | 684 | 563(36.5) | 94(6.1) ^*^ | 32(2.1) | 3(0.2) | 1(0.1) | 0(0) | 0(0) | 0(0) | 0(0) | 0(0) | 0(0) | 0(0) | 0(0) |
|  | 2nd year | 508 | 584(37.8) | 97(6.3) ^*^ | 9(0.6) | 1(0.1) | 1(0.1) | 1(0.1) | 0(0) | 0(0) | 0(0) | 0(0) | 0(0) | 0(0) | 0(0) |
| Cardiovascular Medicine | Total | 4756 | 562(40.5) | 483(34.8) ^*^ | 277(20.0) | 34(2.5) | 5(0.4) | 2(0.1) | 2(0.1) | 0(0) | 1(0.1) | 0(0) | 0(0) | 0(0) | 0(0) |
|  | 1st year | 3356 | 140(9.1) | 300(19.4) ^*^ | 224(14.5) | 27(1.7) | 1(0.1) | 0(0) | 1(0.1) | 0(0) | 0(0) | 0(0) | 0(0) | 0(0) | 0(0) |
|  | 2nd year | 1400 | 442(28.6) | 183(11.9) ^*^ | 53(3.4) | 7(0.5) | 4(0.3) | 2(0.1) | 1(0.1) | 0(0) | 1(0.1) | 0(0) | 0(0) | 0(0) | 0(0) |
| Endocrinology Metabolism | Total | 2748 | 839(60.5) | 424(30.6) ^*^ | 111(8.0) | 7(0.5) | 5(0.4) | 0(0) | 0(0) | 0(0) | 0(0) | 0(0) | 0(0) | 0(0) | 0(0) |
|  | 1st year | 1756 | 357(23.1) | 245(15.9) ^*^ | 82(5.3) | 6(0.4) | 3(0.2) | 0(0) | 0(0) | 0(0) | 0(0) | 0(0) | 0(0) | 0(0) | 0(0) |
|  | 2nd year | 992 | 482(31.2) | 179(11.6) ^*^ | 29(1.9) | 1(0.1) | 2(0.1) | 0(0) | 0(0) | 0(0) | 0(0) | 0(0) | 0(0) | 0(0) | 0(0) |
| Gastroenterology | Total | 5152 | 586(42.3) | 462(33.3) ^*^ | 267(19.3) | 34(2.5) | 17(1.2) | 3(0.2) | 13(0.9) | 3(0.2) | 1(0.1) | 0(0) | 0(0) | 0(0) | 0(0) |
|  | 1st year | 3500 | 163(10.6) | 278(18.0) ^*^ | 210(13.6) | 22(1.4) | 5(0.3) | 1(0.1) | 12(0.8) | 2(0.1) | 0(0) | 0(0) | 0(0) | 0(0) | 0(0) |
|  | 2nd year | 1652 | 423(27.4) | 184(11.9) ^*^ | 57(3.7) | 12(0.8) | 12(0.8) | 2(0.1) | 1(0.1) | 1(0.1) | 1(0.1) | 0(0) | 0(0) | 0(0) | 0(0) |
| General Internal Medicine | Total | 3620 | 821(59.2) | 350(25.3) ^*^ | 149(10.8) | 41(3.0) | 10(0.7) | 4(0.3) | 8(0.6) | 0(0) | 1(0.1) | 1(0.1) | 0(0) | 0(0) | 0(0) |
|  | 1st year | 2248 | 368(23.8) | 177(11.5) ^*^ | 102(6.6) | 29(1.9) | 6(0.4) | 1(0.1) | 8(0.6) | 0(0) | 1(0.1) | 1(0.1) | 0(0) | 0(0) | 0(0) |
|  | 2nd year | 1372 | 453(29.3) | 173(11.2) ^*^ | 47(3.0) | 12(0.8) | 4(0.3) | 3(0.2) | 0(0) | 0(0) | 0(0) | 1(0.1) | 0(0) | 0(0) | 0(0) |
| Hematology | Total | 1680 | 1044(75.3) | 271(19.6) ^*^ | 66(4.8) | 4(0.3) | 0(0) | 1(0.1) | 0(0) | 0(0) | 0(0) | 0(0) | 0(0) | 0(0) | 0(0) |
|  | 1st year | 1128 | 472(30.6) | 167(10.8) ^*^ | 49(3.2) | 4(0.3) | 0(0) | 1(0.1) | 0(0) | 0(0) | 0(0) | 0(0) | 0(0) | 0(0) | 0(0) |
|  | 2nd year | 552 | 572(37.0) | 104(6.7) ^*^ | 17(1.1) | 0(0) | 0(0) | 0(0) | 0(0) | 0(0) | 0(0) | 0(0) | 0(0) | 0(0) | 0(0) |
| Infectious Diseases | Total | 536 | 1265(91.3) | 112(8.1) ^*^ | 7(0.5) | 0(0) | 2(0.1) | 0(0) | 0(0) | 0(0) | 0(0) | 0(0) | 0(0) | 0(0) | 0(0) |
|  | 1st year | 200 | 651(42.2) | 36(2.3) ^*^ | 5(0.3) | 0(0) | 1(0.1) | 0(0) | 0(0) | 0(0) | 0(0) | 0(0) | 0(0) | 0(0) | 0(0) |
|  | 2nd year | 336 | 614(39.8) | 76(4.9) ^*^ | 2(0.1) | 0(0) | 1(0.1) | 0(0) | 0(0) | 0(0) | 0(0) | 0(0) | 0(0) | 0(0) | 0(0) |
| Nephrology | Total | 2680 | 846(61.0) | 424(30.6) ^*^ | 103(7.4) | 12(0.9) | 1(0.1) | 0(0) | 0(0) | 0(0) | 0(0) | 0(0) | 0(0) | 0(0) | 0(0) |
|  | 1st year | 1544 | 389(25.2) | 229(14.8) ^*^ | 69(4.5) | 5(0.3) | 1(0.1) | 0(0) | 0(0) | 0(0) | 0(0) | 0(0) | 0(0) | 0(0) | 0(0) |
|  | 2nd year | 1136 | 457(29.6) | 195(12.6) ^*^ | 34(2.2) | 7(0.5) | 0(0) | 0(0) | 0(0) | 0(0) | 0(0) | 0(0) | 0(0) | 0(0) | 0(0) |
| Neurology | Total | 2544 | 875(63.1) | 397(28.6) ^*^ | 103(7.4) | 11(0.8) | 0(0) | 0(0) | 0(0) | 0(0) | 0(0) | 0(0) | 0(0) | 0(0) | 0(0) |
|  | 1st year | 1564 | 387(25.1) | 227(14.7) ^*^ | 73(4.7) | 6(0.4) | 0(0) | 0(0) | 0(0) | 0(0) | 0(0) | 0(0) | 0(0) | 0(0) | 0(0) |
|  | 2nd year | 980 | 488(31.6) | 170(11.0) ^*^ | 30(1.9) | 5(0.3) | 0(0) | 0(0) | 0(0) | 0(0) | 0(0) | 0(0) | 0(0) | 0(0) | 0(0) |
| Respiratory | Total | 3488 | 732(52.8) | 463(33.4) ^*^ | 169(12.2) | 19(1.4) | 2(0.1) | 0(0) | 1(0.1) | 0(0) | 0(0) | 0(0) | 0(0) | 0(0) | 0(0) |
|  | 1st year | 2376 | 266(17.2) | 274(17.7) ^*^ | 139(9) | 14(0.9) | 0(0) | 0(0) | 0(0) | 0(0) | 0(0) | 0(0) | 0(0) | 0(0) | 0(0) |
|  | 2nd year | 1112 | 466(30.2) | 189(12.2) ^*^ | 30(1.9) | 5(0.3) | 2(0.1) | 0(0) | 1(0.1) | 0(0) | 0(0) | 0(0) | 0(0) | 0(0) | 0(0) |
| Other Internal Medicine | Total | 680 | 1264(91.2) | 85(6.1) ^*^ | 31(2.2) | 3(0.2) | 1(0.1) | 2(0.1) | 0(0) | 0(0) | 0(0) | 0(0) | 0(0) | 0(0) | 0(0) |
|  | 1st year | 344 | 632(40.9) | 39(2.5) ^*^ | 20(1.3) | 1(0.1) | 1(0.1) | 0(0) | 0(0) | 0(0) | 0(0) | 0(0) | 0(0) | 0(0) | 0(0) |
|  | 2nd year | 336 | 632(40.9) | 46(3.0) ^*^ | 11(0.7) | 2(0.1) | 0(0) | 2(0.1) | 0(0) | 0(0) | 0(0) | 0(0) | 0(0) | 0(0) | 0(0) |

^*^Rotation period with the largest number of people in each department (excluding no rotation)

**PYG: Postgraduate year**

**RPP index: Resident physician popularity index**
